# Neurosurgical Outcomes of Isolated Hemorrhagic Mild Traumatic Brain Injury

**DOI:** 10.7759/cureus.5982

**Published:** 2019-10-24

**Authors:** Evan M Krueger, Matthew Putty, Michael Young, Brandon Gaynor, Ellen Omi, Hamad Farhat

**Affiliations:** 1 Neurosurgery, Advocate Health Care, Downers Grove, USA; 2 Neurosurgery, Advocate Health Care, Normal, USA; 3 Neurosurgery, Advocate Bromenn Medical Center, Normal, USA; 4 Neurosurgery, Advocate Christ Medical Center, Oak Lawn, USA; 5 Trauma Surgery, Advocate Health Care, Oak Lawn, USA

**Keywords:** mild traumatic brain injury, subdural hematoma, neurosurgical intervention, epidural hematoma, neurocritical care

## Abstract

Introduction

Mild traumatic brain injury (TBI) is common but its management is variable.

Objectives

To describe the acute natural history of isolated hemorrhagic mild TBI.

Methods

This was a single-center, retrospective chart review of 661 patients. Inclusion criteria were consecutive patients with hemorrhagic mild TBI. Exclusion criteria were any other acute traumatic injury and significant comorbidities. Variables recorded included neurosurgical intervention and timing, mortality, emergency room disposition, intensive care unit (ICU) length of stay (LOS), discharge disposition, repeat computed tomography head (CTH) indications and results, neurologic exam, age, sex, Glasgow Coma Scale (GCS) score, and hemorrhage type.

Results

Overall intervention and unexpected delayed intervention rates were 9.4% and 1.5%, respectively. The mortality rate was 2.4%. A 10-year age increase had 26% greater odds of intervention (95% CI, 9.6-45%; P<.001) and 53% greater odds of mortality (95% CI, 11-110%; P=.009). A one-point GCS increase had 49% lower odds of intervention (95% CI, 25-66%; P<.001) and 50% lower odds of mortality (95% CI, 1-75%; P=.047). Subdural and epidural hemorrhages were more likely to require intervention (P=.02). ICU admission was associated with discharge to an acute care facility (OR, 2.9; 95% CI, 1.4-6.0; P=.003). Neurologic exam changes were associated with a worsened CTH scan (OR, 12.3; 95% CI, 7.0-21.4; P<.001) and intervention (OR, 15.1; 95% CI, 8.4-27.2; P<.001).

Conclusions

Isolated hemorrhagic mild TBI patients are at a low, but not clinically insignificant, risk of intervention and mortality.

## Introduction

Traumatic brain injury (TBI) is a common and costly disease. The annual incidence of traumatic TBI in the United States is 1.6 million, resulting in 290,000 hospitalizations [1]. Approximately 95% have mild TBI, defined as a Glasgow Coma Scale (GCS) score of 13-15 [2]. In 2010, the yearly economic burden in the United States for non-fatal TBI was nearly $88 billion [3]. The intensive care unit (ICU) cost for Medicare rose 36% from 1994-2004, with an average cost per day of $2,574 [4]. ICU beds represent 8% of United States hospital beds but are responsible for 28% of acute hospital charges [5].

Care for mild TBI patients is highly variable [6]. Alternatively, prognostic models exist for moderate and severe TBI patients [7-9]. Furthermore, guidelines focus specifically on severe TBI patients [10]. Therefore, there is an opportunity to improve the consistency, quality, and safety of mild TBI triage criteria and acute management. Evidence-based decision-support tools could help improve patient outcomes and resource utilization.

The purpose of this study was to describe the acute natural history of isolated hemorrhagic mild TBI. The primary objective was to determine the risk of neurosurgical intervention and mortality. The secondary objectives were to examine modifiable care costs - emergency department (ED) disposition, ICU length of stay (LOS), computed tomography head (CTH) scans, and neurologic exam - pertaining to short-term outcomes. The tertiary objectives were to determine if demographic and clinical variables - age, sex, GCS, and hemorrhage type - were predictive of hospital course.

## Materials and methods

Inclusion criteria

This was a single-center, retrospective chart review that obtained institutional review board (IRB) approval (IRB #00001341). All consecutive patients from the institutional trauma registry between January 1, 2011, and December 31, 2016, were queried. Inclusion criteria were: age ≥ 18 years, mild TBI defined as GCS ≥13, and any acute intracranial hemorrhage on CTH scan upon arrival.

Exclusion criteria

Any other acute traumatic injury or injury severity scale >25; open or penetrating intracranial wound; skull fractures; suspected or confirmed cerebrospinal fluid leak; and significant pre-existing comorbidities defined as cirrhosis, end-stage renal disease, dementia, previous stroke, and any type of previous TBI.

Study variables

The neurosurgical intervention (craniectomy, craniotomy, burr hole, external ventricular drain (EVD), or intracranial pressure monitor placement) was recorded. The neurosurgical intervention and mortality patients were combined for a subset analysis, recording the anti-coagulation use, anti-coagulation reversal agents, injury mechanism (ground-level falls, falls greater than ground level, motor vehicle collision, assault, or other), timing of intervention (immediately upon emergency department (ED) disposition, planned urgently within 24 hours, or unexpected/delayed), indications for intervention (radiographic or neurologic exam), and cause of mortality (neurologic or non-neurologic). Modifiable care cost variables for the entire study sample included ED disposition (operating room, ICU, step-down unit, floor unit, ED observation unit, or direct discharge), ICU LOS, and discharge disposition (home, acute care facility, or rehabilitation unit). Patients discharged to police custody, home health, or their original nursing facility were categorized as home. Patients discharged to a psychiatric facility, new nursing home, or new skilled nursing facility were categorized as acute care facility. Patients discharged to hospice were categorized as rehabilitation unit. Additional modifiable care cost variables included whether repeat CTH scans were obtained, indications for repeat scans (routine: done after a subjective period of time or selective: due to change in neurologic exam) and the results of scans (stable, improved, or worsening); and if there was a change in neurologic exam (a drop in GCS by ≥2 points, anisocoria, focal neurologic deficit, seizure, worsening/severe headache, nausea, or vomiting). Demographic and clinical variables recorded for the entire sample size were age, sex, GCS on presentation, and type of acute intracranial hemorrhage (epidural hematoma (EDH), subdural hematoma (SDH), subarachnoid hemorrhage (SAH), intraparenchymal hemorrhage (IPH), or intraparenchymal contusion (IPC)).

Statistical analysis

A sample of convenience was utilized. An a priori power analysis was not performed since consecutive charts were reviewed. Logistic regression models were used to model the type of neurosurgical intervention and mortality versus age, sex, GCS, and hemorrhage type. Firth’s bias correction was used in all logistic regression models as needed. A student’s t-test was used to compare the mean ages between survivors and non-survivors. Within the neurosurgical intervention and mortality patients combined subset, a Pearson’s correlation test was used to examine the association with anti-coagulation medicine, anti-coagulation reversal agent, mechanism of injury, and type of neurosurgical intervention. Generalized logit models were used to model the ED disposition versus age, sex, GCS, and hemorrhage type. To assess association with ED disposition and hemorrhage type and avoid quasi-complete separation of data points, hemorrhage types were collapsed into groups (Group 1: EDH or SDH; and Group 2: IPC, IPH, SAH, or multiple). Generalized linear models with a negative binomial distribution and log link were used to model ICU LOS versus age, sex, GCS, and hemorrhage type. A generalized logit model was used to model ED disposition versus discharge disposition. To assess association with ED disposition and discharge disposition and avoid quasi-complete separation of data points, ED disposition was collapsed into groups (Group 1: floor, step-down, and ED observation; Group 2: ICU; and Group 3: operating room). Generalized linear models with a negative binomial distribution and log link were used to model CTH results versus age, sex, GCS, and hemorrhage type. A Wilcox 2 sample t-test was used to compare median ICU LOS for those with stable or worsening CTH scans. Logistic regression models were used to model the neurologic exam versus age, sex, GCS, and hemorrhage type. An unadjusted chi-square test was used to compare the neurologic exam with neurosurgical intervention. Data were managed using REDCap (Tennessee, US) [11]. Analyses were performed using SAS (version 9.4; SAS Institute Inc, North Carolina, US). Odds ratio (OR) with 95% confidence intervals (CI), and mean ± 1 standard deviation are reported. P<.05 was considered statistically significant.

## Results

Patient characteristics

A total of 4,016 charts were screened, and 661 met inclusion criteria. The study sample is described in Table 1.

**Table 1:** Study sample demographic and clinical variables STD, standard deviation; M, male; F, female; GCS, Glasgow Coma Scale; EDH, epidural hematoma; SDH, subdural hematoma; SAH, subarachnoid hemorrhage; IPH, intraparenchymal hemorrhage; IPC, intraparenchymal contusion

Age	Sex	GCS	Hemorrhage
Mean 60.5	M: 60.2% (398/661)	13: 4.8% (32/661)	EDH: 1.4% (9/661)
Range 18-98	F: 39.8% (263/661)	14: 18.8% (124/661)	SDH: 38.3% (253/661)
STD 21.7		15: 76.4% (505/661)	SAH: 27.4% (181/661)
			IPH: 10.1% (67/661)
			IPC: 9.1% (60/661)
			Multiple: 13.8% (91/661)

Neurosurgical intervention patients

Those requiring neurosurgical intervention are described in Table 2. Intervention was performed in 9.4% (62/661) of patients. The most common intervention was a craniotomy (69.3%, 43/62). Of the entire sample size, 6.1% (40/661) had an intervention emergently after ED disposition, 1.8% (12/661) had a planned urgent procedure within 24 hours, and 1.5% (10/661) had an unexpected delayed intervention. Of these 10 delayed intervention patients, the indications were: 70% (7/10) for a neurologic exam change, 20% (2/10) for radiographic worsening found on routine CTH scans without a neurologic change, and 10% (1/10) where no indication was recorded.

Those requiring neurosurgical intervention were significantly older (69 (±17.8) vs 60 (±21.9) years old; P<.001). A 10-year increase in age was associated with a 26% greater odds of intervention (95% CI, 9.6-45%; P<.001). There was no association between sex and intervention (P=.65). A one-point increase in GCS was associated with a 49% lower odds for intervention (95% CI, 25-66%; P<.001). Of those undergoing intervention, the most common hemorrhage type was SDH (79%, 49/62). Of the SAH types, 0.55% (1/181) required an intervention (an EVD placed after a delayed neurologic deterioration). SDH and EDH were more likely to require intervention as compared to all other hemorrhage types (P=.02).

**Table 2:** Neurosurgical intervention patients Neurosurgical intervention timing was either immediately upon emergency room disposition, planned urgently within 24 hours, or unexpected delayed. EVD, external ventricular drain; BOLT, intracranial pressure monitor device; EDH, epidural hematoma; SDH, subdural hematoma; SAH, subarachnoid hemorrhage; IPH, intraparenchymal hemorrhage; IPC, intraparenchymal contusion

Intervention	Timing	Hemorrhage
Craniectomy: 11.3% (7/62)	Immediate: 64.5% (40/62)	EDH: 4.8% (3/62)
Craniotomy: 69.4% (43/62)	Planned: 19.7% (12/62)	SDH: 79.0% (49/62)
Burr Hole: 17.7% (11/62)	Delayed: 16.4% (10/62)	SAH: 1.61% (1/62)
EVD: 1.6% (1/62)		IPH: 6.5% (4/62)
BOLT: 0% (0/62)		IPC: 0% (0/62)
		Multiple: 8.1% (5/62)

Mortality patients

The mortality rate was 2.4% (16/661). Survivors were significantly younger (60 (±21.7) vs 75 (±15.4) years old; P=.009). A 10-year increase in age was associated with 53% greater odds of mortality (95% CI, 11-110%; P=.009). There was no association between mortality and sex (P=.48). A one-point increase in GCS was associated with 50% lower odds of mortality (95% CI, 1-75%; P=.047). There was no association between mortality and hemorrhage type (P=.64). Of the 16 mortalities, 50% (8/16) underwent neurosurgical intervention. The cause of death was attributed to neurologic (68.8%, 11/16) or non-neurologic (32.2%, 5/16) reasons.

Combined intervention and mortality subset

The combined neurosurgical (n=62) and non-neurosurgical mortality (n=8) patients subset is described in Table 3. Within this subset, there were no significant associations between anti-coagulation medicine, anti-coagulation reversal agent, mechanism of injury, and type of neurosurgical intervention (all P>.05).

**Table 3:** Combined neurosurgical intervention and mortality patient subset Fall was defined as greater than ground level. ASA, aspirin; X Inhibitory, factor X inhibitor; Thrombin, direct thrombin inhibitor; NSAID, non-steroidal anti-inflammatory drug; Vit K, vitamin K; FFP, fresh frozen plasma; PCC, prothrombin complex concentrate; Cryo, cryoprecipitate; VII, factor VII; Plts; platelets; GLF, ground-level fall; MVC, motor vehicle collision

Anti-Coagulation	Reversal Agents	Mechanism of Injury
ASA: 22.9% (16/70)	Vit K: 7.1% (5/70)	GLF: 72.9% (51/70)
Plavix: 7.1% (5/70)	FFP: 12.9% (9/70)	Fall: 8.6% (6/70)
Coumadin: 8.6% (6/70)	PCC: 4.3% (3/70)	MVC: 7.1% (5/70)
X Inhibitor: 1.4% (1/70)	Cryo: 1.4% (1/70)	Assault: 7.1% (5/70)
Thrombin: 0% (0/70)	VII: 0% (0/70)	Other: 4.3% (3/70)
NSAID: 0% (0/70)	Plts: 11.4% (8/70)	
None: 64.3% (45/70)	Other: 0% (0/70)	
	None: 75.7% (53/70)	

ED disposition

Modifiable care cost variables are described in Table 4. For ED disposition, a 10-year increase in age was associated with a 15% lower odds of floor compared to ICU admission (95% CI, 5-24%; P<.001), and a 16% lower odds of ED observation unit compared to ICU admission (95% CI, 2-28%; P<.001). Sex (P=.32) and GCS (P=.10) were not associated with ED disposition. SDH and EDH (Group 1) were associated with a 45% lower odds of floor admission compared to ICU admission (95% CI, 5-68%; P<.001) and a 77% lower odds of ED observation unit compared to ICU admission (95% CI, 39-91%; P<.001).

ICU LOS

The mean ICU LOS was 2.1 (±2.3) days. A 10-year increase in age was associated with a 5% increase in ICU LOS (95% CI, 2-9%; P=.004). There was no correlation between sex and ICU LOS (P=.12). A one-point increase in GCS was associated with a 23% lower length of ICU LOS (95% CI, 14-31%; P<.001). SDH (2.5 (±2.6) days; 95% CI, 2.2-2.7) and multiple hemorrhage (2.8 (±3.2) days; 95% CI, 2.4-3.3) types were associated with longer ICU stays compared to IPC (1.7 (±1.2) days; 95% CI, 1.3-2.2), IPH (1.8 (±1.1) days; 95% CI,1.4-2.3), and SAH (1.5 (±1.4) days; 95% CI, 1.3-1.8) (all P<.02).

Discharge disposition

For discharge disposition, the odds of acute care facility to home was higher for those admitted to the ICU (Group 2) as compared to those admitted to the floor/step-down/ED observation unit (Group 1) (OR, 2.9; 95% CI, 1.4-6.0; P=.003).

**Table 4:** Modifiable care cost variables ED disposition was either to the operating room, ICU, step-down unit, floor unit, ED observation unit, or direct discharge. Discharge was either to home, acute care facility, or rehabilitation unit. ED, emergency department; ICU, intensive care unit; ED Obs, ED observation unit; DC, discharge; LOS, length of stay; STD, standard deviation

ED disposition	ICU LOS	Discharge
Operating Room: 6.1% (40/661)	mean: 2.1	Home: 71.3% (471/661)
ICU: 77% (509/661)	range: 1-25	Acute Care: 19.8% (131/661)
Step-Down: 1.2% (8/661)	STD: 2.0	Rehab: 6.5% (43/661)
Floor: 11.0% (73/661)		Morgue: 2.4% (16/661)
ED Obs: 5.4% (36/661)		
DC: 0% (0/661)		

Imaging

The CTH scan and neurologic exam variables are listed in Table 5. The odds of a worsening CTH scan were 12.3 times greater with a neurologic exam change as compared to a stable neurologic exam (95% CI, 7.0-21.4; P<.001). A stable CTH scan was associated with a shorter ICU LOS as compared to radiographic worsening (median 1 vs 3 days; P<.001).

Neurologic exam

Of the 11.4% (75/661) of patients with a neurologic change, 46.7% (35/75) had worsening CTH scans while 53.3% (40/75) had stable scans. Age (P=.84) and sex (P=.06) were not associated with a neurologic change. A one-point increase in GCS was associated with a 46% lower odds of a neurologic change (95% CI, 22.0-63.0%; P=.001). SDH (OR, 4.82; 95% CI, 2.05-11.36; P<.001) and multiple hemorrhages (OR, 6.34; 95% CI, 2.47-16.29; P<.001) were associated with higher odds of a neurologic change as compared to SAH. A neurologic exam change was significantly associated with a neurosurgical intervention (OR, 15.1; 95% CI, 8.4-27.2; P<.001).

**Table 5:** Repeat imaging and neurologic exam Imaging was obtained either routinely or for a change in the neurologic exam defined as a drop in Glasgow Coma Scale score by ≥2 points, anisocoria, focal neurologic deficit, seizure, worsening/severe headache, nausea, or vomiting. EDH, epidural hematoma; SDH, subdural hematoma; SAH, subarachnoid hemorrhage; IPH, intraparenchymal hemorrhage; IPC, intraparenchymal contusion

Repeat Imaging	Reason for Imaging	Exam	Worsened Exam
Improved: 8.9% (59/661)	Routine: 88.7% (586/661)	Stable: 88.7% (586/661)	EDH: 0% (0/9)
Stable: 79.4% (528/661)	Neuro change: 11.3% (75/661)	Worse: 11.3% (75/661)	SDH: 11.5% (29/253)
Worse: 11.2% (74/661)			SAH: 3.9% (7/181)
			IPH: 16.4% (11/67)
			IPC: 8.3% ( 5/60)
			Multiple: 24.2% (22/91)

## Discussion

Our primary objective was to determine the risk of neurosurgical intervention and mortality. The overall intervention and mortality rates were 9.4% and 2.4%, respectively. In our experience, delayed deterioration necessitating neurosurgical intervention on initially non-surgical patients is a feared complication that often influences decisions to obtain neurosurgical consultation, repeat imaging, and admit to higher levels of care, despite limited evidence to suggest benefit. In this study, only 1.5% of patients had delayed deterioration requiring intervention, and, furthermore, only 0.3% had an unplanned delayed intervention that was indicated solely on a routine CTH scan without a neurologic change. Slightly higher (3.8%) delayed intervention rates in non-isolated hemorrhagic mild TBI have been reported [12]. A meta-analysis of 46 hemorrhagic mild TBI studies (n=65,724) showed neurosurgical intervention and mortality rates of 3.5% and 1.4%, respectively [13]. In the current study, institutional bias and heterogeneous samples may have contributed to our reported higher neurosurgical intervention rate. Furthermore, unlike other studies, we choose to examine isolated hemorrhagic mild TBI in patients without significant comorbidities. These patients may have had more favorable surgical risk-benefit profiles to justify intervention.

Our secondary objective was to examine modifiable care costs pertaining to short-term outcomes. We found patients admitted to the ICU were 2.9 times more likely to be discharged to an acute care facility. The most plausible explanations are ICU admission criteria or hospital course complications not measured in our study since we excluded significant comorbidities. This does, however, question whether ICU admission improves outcomes compared to less resource-demanding units for this patient population. Other multicenter, prospective studies have shown no differences in six-month outcomes for admission to various levels of care [14]. A single-center, prospective study reported no differences in mortality, operative intervention, or discharge disposition for ICU versus step-down unit admission [15]. Ultimately, there are no clear recommendations when ICU admission is warranted, mainly because of low-quality evidence and the difficulty of generalizing criteria to institutions with variable resources and capabilities.

The utility of routine repeat imaging in favor of selective imaging done for a change in the neurologic exam is debatable. In support of routine imaging, obtaining a stable CTH scan was associated with a significantly shorter ICU LOS in our study. There may have been an additional clinical benefit of routine repeat imaging not measured in our study such as providing justification for anti-coagulation medication or discharge. Stable repeat imaging may also be useful for medical liability coverage. However, we feel our data more strongly support selective imaging. Only 11.2% of all patients had worsened CTH scans. A change in the neurologic exam was associated with 12.3 times greater odds of worsening CTH scans and 15.1 times greater odds for neurosurgical intervention. Others have reported that selective imaging is more predictive for intervention than routine imaging [16-18]. It may be difficult to justify the fiscal costs of routine imaging since this has not been shown to be significantly cost-effective [19]. Further data is needed to confirm whether a thorough neurologic exam is a more efficacious, cost-effective, and safe way to follow these patients compared to routine serial imaging, as well as the appropriate duration of neurologic exam monitoring to avoid adverse events.

Our tertiary objective was to examine if demographic and clinical variables were predictive of hospital course. In our study, a 10-year increase in age was associated with a 5% increase in ICU LOS, 26% greater odds of neurosurgical intervention, and 53% greater odds of mortality. Age has previously been reported as a predictor of clinical decline, neurosurgical intervention, and mortality [13,18,20]. Because we assigned age as a continuous variable, we did not identify an age-specific inflection point that would dictate a specific set of outcomes, for example, defining a specific age cutoff point to justify ICU admission because of increased risk.

In our study, GCS was found to be predictive of hospital course and neurosurgical intervention. A one-point increase in GCS was associated with a 23% lower ICU LOS, 46% lower odds of neurologic change, 49% lower odds of intervention, and 50% lower odds of mortality. GCS has previously been reported as a predictor of neurologic decline, intervention, and mortality [13,18,21].

We found heterogeneity amongst different hemorrhages. SDH and EDH were significantly more likely to be admitted to the ICU. SDH and multiple hemorrhages were the most likely to have a neurologic change as compared to SAH. The rates of neurosurgical intervention for SDH and EDH were significantly higher than for other hemorrhage types at 19.4% and 33%, respectively. SDH and EDH have previously been reported to be the most likely hemorrhage subtypes associated with adverse events and intervention [13,18,21]. In contrast, for SAH, our intervention rate was only 0.6%. Larger studies of isolated traumatic SAH have shown intervention rates of 0.0017% and 0.24% [22-23]. The heterogeneous clinical course of different hemorrhage types suggests that merely the presence of intracranial hemorrhage may not be sufficient to justify costly management decisions such as ICU admission.

There are limitations to the present study. First, this was a single-center retrospective study. Second, although the majority of patients were GCS 15, charts were reviewed consecutively and should be free of sampling bias. Third, EDH was underrepresented in our sample most likely because we excluded skull fractures. Next, our sample size of isolated hemorrhagic mild TBI may not be applicable to poly-system trauma patients. Last, we did not obtain long-term follow-up, which may have identified patients who had very delayed neurosurgical intervention or had hospital readmission. Nonetheless, our study provides valuable insight. Our large sample size of isolated hemorrhagic mild TBI in patients without significant comorbidities is not well-described in the literature.

Others have explored cost-saving protocols for mild TBI. Pruitt et al. 2017 describe a single-center, retrospective study where low-risk patients were identified for ED observation; none of which deteriorated clinically or returned to the ED [24]. Yun et al. (2017) designed a single-center, prospective study for low-risk hemorrhagic mild TBI patients that showed safety efficacy and reduced admission rates [25]. Clearly identifying low-risk hemorrhagic mild TBI patients at initial triage may be an important branch point during decision-making and represent an opportunity to improve care and lower costs.

## Conclusions

In summary, we performed a single-center, retrospective chart review of isolated hemorrhagic mild TBI. Patients were at a low, but not clinically insignificant, risk of neurosurgical intervention and mortality. ICU admission was associated with increased odds of discharge to an acute care facility. The neurologic change was associated with a worsening CTH scan and need for intervention. Age, GCS, and hemorrhage type were predictive of hospital course. There is no standard of care for mild TBI patients, despite representing the majority of all TBI. Physician judgment will ultimately be the most important decision-making factor, but it should be evidence-based. Developing a widely applicable absolute management scheme that captures all the nuances of hemorrhagic mild TBI is challenging. Using these data, future carefully selected patients based on age, GCS, and hemorrhage type could benefit from streamlined care.
